# Umbilical hernia alloplastic dual-mesh treatment in cirrhotic patients

**Published:** 2013-03-25

**Authors:** RE Guriță, F Popa, C Bălălău, RV Scăunașu

**Affiliations:** * General Surgery Department, "Sf. Pantelimon" Emergency Hospital, Bucharest; ** "Carol Davila" University of Medicine and Pharmacy, Bucharest; General Surgery Department, "Sf. Pantelimon" Emergency Hospital, Bucharest; *** General Surgery Department, "Colțea" Clinical Hospital, Bucharest

**Keywords:** complicated umbilical hernia, cirrhosis, alloplastic intraperitoneal repair

## Abstract

Rationale. Abdominal wall hernias represent a pathology with an impressive prevalence among the population of patients with cirrhosis complicated by ascites.

The aggressive surgical approach of umbilical hernia for patients with cirrhotic background remains a controversial problem, accompanied by anesthetic and surgical risk. Its indication remains fully justified in case of severe symptoms or life threatening complications: strangulation, incarceration, evisceration.

Objective. This article evaluates results obtained by using dual-mesh alloplastic materials for surgical treatment of umbilical hernias affecting cirrhotic patients with incipient liver injury.

Methods and Results. Our lot consists of twelve patients with ages between 45 and 65 years, diagnosed with hepatic cirrhosis, without other associated comorbidities. All patients were admitted for strangulated umbilical hernia.

Among the analyzed lot, no decease was encountered, the morbidity being limited to two cases of parietal suppuration, solved conservatively, without the mesh removal. There were no ascitic fistulas. No recurrences were registered for a 12 months tracking period.

Discussion. The presence of cirrhosis implies a high anesthetic and surgical risk, the intervention being grafted by a substantial increase of mortality and morbidity in an emergency setting.

The development of new alloplastic materials, together with the modern anesthetic techniques, allows superior results for patients with incipient hepatic injury.

## Introduction

Abdominal wall hernias represent a pathology with an impressive prevalence among the population of patients with cirrhosis complicated by ascites.

 Among these, the umbilical hernias are the most frequent, occurring in more than 20% of the cases [**9,13,18**]. The imbalance between the increased abdominal pressure, caused by the formation of ascites, and the weakening of the fascia and muscles of the abdominal wall, a consequence of the hipoproteic status, both contribute to the widening of the umbilical ring.

 Elective surgical repair of umbilical hernias in cirrhotic patients prevents complications (strangulation), but it is often discouraged by the high morbidity and mortality rates, and by the increased recurrence of hernia. For this type of patients, the conservative attitude is followed by the rapid evolution of the disease [**2,3,9**]. As the hernias grow in size, the fast distension of the tissues causes ischemic lesions, ulcers and fistulas or development of the secondary bacterial peritonitis.

 The aggressive surgical approach of umbilical hernia for patients with cirrhotic background remains a controversial problem, accompanied by anesthetic and surgical risk. Its indication remains fully justified in case of severe symptoms, life threatening complications: strangulation, incarceration, evisceration or severe trophic disorders [**4,5**].

 A series of specific complications can occur after umbilical hernia surgery: ascitic fistula, renal failure, abdominal wall infections, hepatic failure and hernia recurrence. Post-operative mortality in cirrhotic patients is correlated with the severity of liver function impairment, appreciated using Child-Pugh and MELD scores [**6-8,10**].

## Material and Method

 Historically, the alloplastic treatment of umbilical hernias for cirrhotic patients was avoided, invoking the high risk of mesh contamination or fistula development. Recent studies demonstrated a low morbidity and mortality rate for patients with incipient hepatic disease. 

 A key point in the management of the cirrhotic patient treatment is the bidirectional relationship between liver and anesthesia. The anesthetic drugs can interfere with the hepatic metabolism, by decreasing the sanguine flow towards the liver and direct toxicity. In return, as the biotransformation of the majority of used anesthetics is mainly hepatic, the liver with chronic or acute suffering determines an altered answer to the administrated anesthetics [**4,14**]. 

 Our lot consists of twelve patients with ages between 45 and 65 years, diagnosed with hepatic cirrhosis, without other associated comorbidities. After the preoperative evaluation all patients were classified in class A Child-Pugh with a M.E.L.D score <10, considered to have minimum anesthetic risk, morbidity and mortality. All patients were admitted for strangulated umbilical hernia. Hernia sac content treating, by partial omentectomy, was necessary for three of them. 

 Spinal anesthesia was used for seven patients with >75000 thrombocytes and clotting in normal limits, the rest of the cases requiring general anesthesia. 

 Dual Mesh (ePTFE) alloplastic material was used, which allows the surgeon to realize an intraperitoneal repair, similar as a concept to the laparoscopic approach, avoiding some of its risks and the requirement of general anesthesia, but having similar benefits regarding the reduction of the postoperative pain and the effort capacity improvement [**1,6,17**]. 

 At the beginning of the surgery, the prosthetic material is measured and tailored to exceed with at least 4-5 cm the parietal defect. 

**Fig. 1:**
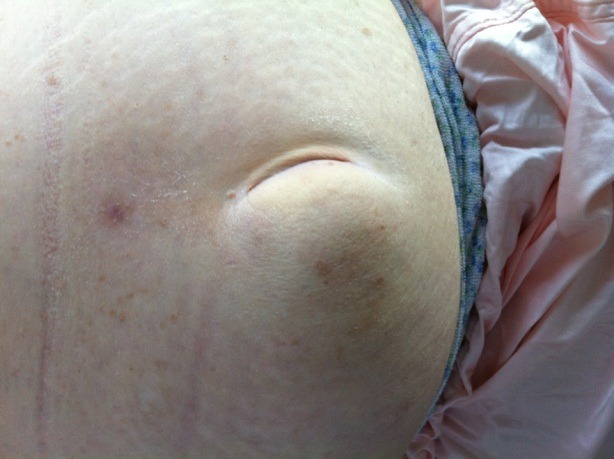
Clinical presentation

**Fig. 2:**
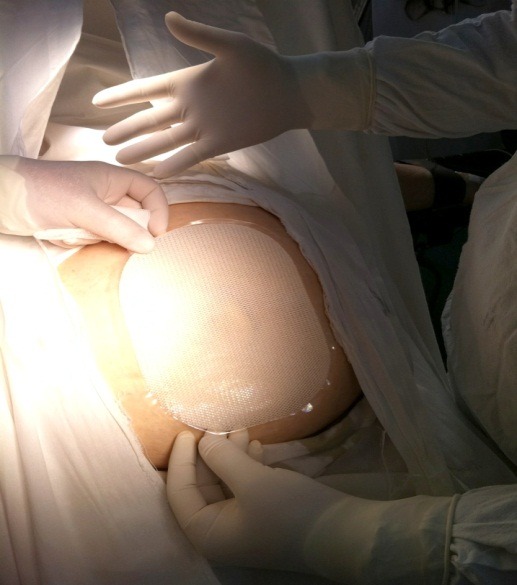
Mesh scaled to the defect dimensions

 Non-resorbable suture stitches are placed circumferential and in the middle of the mesh, far from the parietal defect. Correspondent mini incisions will be realized in the tegument. This will ensure the correct orientation and the fixation of the prosthetic material during the procedure.

**Fig. 3:**
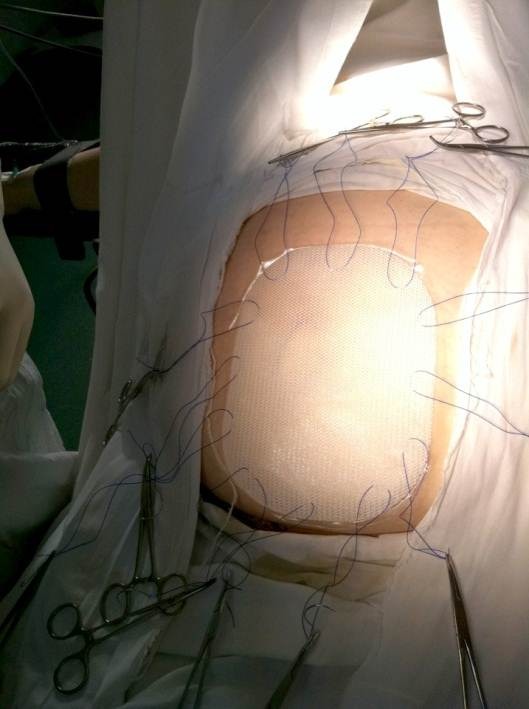
Placement of stitches

 The next step is the dissection of the umbilical hernia sac and treatment of the content when necessary, followed by the preparation of the aponeurotic margins.

**Fig. 5,6,7:**
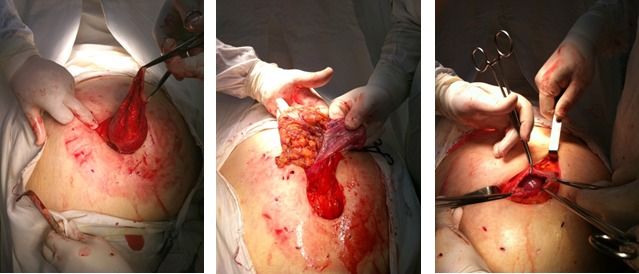
Dissection of the umbilical hernia sac

The mesh fixation stitches are transparietal externalized through the tegumentary mini incisions. Final positioning and knotting are safely and comfortably performed. 

**Fig. 8,9:**
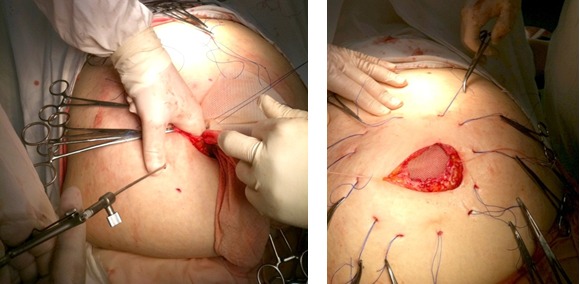
Stitches are extracted by using a laparoscopic suture grasper

The Dual Mesh material is oriented, placed intraperitoneally and secured by the extrategumentary extraction of the stitches at the mini incisions level. For this last time, we used a suture grasper borrowed from the laparoscopic kit, inserted under direct view in order to prevent possible intestinal damage. 

 Abdominal wall is closed in an anatomical fashion, the prosthetic material having a strengthening intent. Drainage was not used.

## Results and discussions

 The presence of cirrhosis implies a high anesthetic and surgical risk, the intervention being grafted by a substantial increase of mortality and morbidity in an emergency setting [**15**]. 

 The developments of new alloplastic materials, together with the modern anesthetic techniques, allow superior results for patients with incipient hepatic injury. 

 Among the analyzed lot, no decease was encountered, the morbidity being limited to two cases of parietal suppuration, conservatively solved, without the mesh removal. There were no ascitic fistulas. No recurrences were registered for a 12 months tracking period. 

 The intervention is followed by fast recovery, similar to minimal invasive laparoscopic techniques and the hospitalization period was between 3 and 5 days. 

 In comparison, the short intervention time, the lack of pneumoperitoneum, the reliable suture and the good quality hemostasis, recommend the open procedure with minimum laparotomy [**12,16**]. 

## Conclusions

1. Disequilibrium between the intra abdominal pressure and the dystrophic wall of the cirrhotic patient frequently causes abdominal wall hernias for this group of patients.

 2. The umbilical hernia is by far the most common, affecting more than 20% of the patients.

 3. The evolution of umbilical hernia is accelerated, often leading to life threatening complications. 

 4. The emergency surgical treatment is followed by an unfavorable prognosis in 40% of the cases [**3,11,18**].

 5. General anesthesia has severe side effects, hepatic and neuro toxicity, thromboembolic risk and decompensation of cirrhosis due to cardiac debit decrease.

 6. For patients without coagulopathy and moderate size hernias, spinal anesthesia can assure the surgical comfort, avoiding these inconveniences.

 7. The use of Dual Mesh with the strengthening intent is a rapid procedure, which provides good quality prosthesis.

 8. The mesh is well tolerated and integrated, because it does not allow the formation of adhesions or the bacterial aggregation in an environment with septic potential through translocation.

 9. By intraperitoneal placing, the wound is protected against ascitic efraction and postoperative complications associated with this (dehiscence, infections and relapse).

 10. The procedure allows surgical treatment of the content for complicated hernias.
